# Salivary MMP-9 Levels in Chronic Periodontitis Patients with Type-II Diabetes Mellitus

**DOI:** 10.3390/molecules27072174

**Published:** 2022-03-28

**Authors:** Romaisa Arshad, Waleed A. Ismail, Batool Zara, Rabia Naseer, Sadia Minhas, Moeez Ansari, Fatema Akhter, Saria A. Khokhar, Abdullah Ali Alqahtani, Huda Abutayyem, Haytham Jamil Alswairki, Mohammad Khursheed Alam

**Affiliations:** 1 Department of Oral Pathology, Shifa College of Dentistry, Shifa Tameer-e-Millat University, Islamabad 44000, Pakistan; romaisa.scd@stmu.edu.pk; 2 Department of Periodontics, Shifa College of Dentistry, Shifa Tameer-e-Millat University, Islamabad 44000, Pakistan; waleed.scd@stmu.edu.pk; 3 Periodontics Unit, School of Dental Sciences, Health Campus, Universiti Sains Malaysia, Kota Bharu 16150, Malaysia; drmoeezansari@gmail.com; 4 Department of Periodontology, College of Dentistry, Foundation University, Islamabad 44000, Pakistan; batool_zara@hotmail.com (B.Z.); saria.arshad@gmail.com (S.A.K.); 5 Department of Oral & Maxillofacial Surgery, Army Medical College, National University of Medical Sciences, Rawalpindi 46000, Pakistan; dr.rabia786@hotmail.com; 6 Department of Oral Pathology, Akhtar Saeed Medical & Dental College, University of Health Sciences, Lahore 53720, Pakistan; sadia.minhas@amdc.edu.pk; 7 Department of Periodontology, Rashid Latif Medical & Dental College, University of Health Sciences, Lahore 53720, Pakistan; 8 Department of Surgical and Diagnostic Sciences, College of Dentistry, Dar Al Uloom University, Riyadh 11512, Saudi Arabia; mstfatima@dau.edu.sa; 9 Department of Conservative Dental Sciences, College of Dentistry, Prince Sattam Bin Abdulaziz University, Alkharj 11942, Saudi Arabia; aa.alqahtani@psau.edu.sa; 10 Center of Medical and Bio-Allied Health Sciences Research, Department of Clinical Sciences, College of Dentistry, Ajman University, Ajman P.O. Box 346, United Arab Emirates; h.abutayyem@ajman.ac.ae; 11 School of Dental Sciences, Universiti Sains Malaysia, Kota Bharu 16150, Malaysia; hitham.swerki@gmail.com; 12 Department of Preventive Dentistry, College of Dentistry, Jouf University, Sakaka 72345, Saudi Arabia; 13 Center for Transdisciplinary Research (CFTR), Saveetha Dental College, Saveetha Institute of Medical and Technical Sciences, Saveetha University, Chennai 600077, India; 14 Department of Public Health, Faculty of Allied Health Sciences, Daffodil lnternational University, Dhaka 1341, Bangladesh

**Keywords:** chronic periodontitis, case-control, diabetes mellitus, matrix metalloproteinase, periodontal parameters

## Abstract

Chronic periodontitis and diabetes mellitus share a two-way relationship, the common factor being the inflammatory-mediated pathway, and various cytokines are released during this inflammatory cascade, one of which being matrix metalloproteinase-9. The aim of this study was to identify whether the levels of matrix metalloproteinase-9 are increased due to type-II diabetes mellitus in chronic periodontitis patients. It was an observational, analytical, case-control study. Thirty subjects were recruited in the test group, who were suffering from type-II diabetes mellitus and chronic periodontitis, and 30 subjects in the control group, who were subjects with chronic periodontitis but systemically healthy. Periodontal parameters, including the plaque score, gingival bleeding index, probing pocket depth and clinical attachment level of the subjects, were measured, saliva samples of all of the subjects were collected and salivary matrix metalloproteinase-9 levels were analyzed by an enzyme-linked immunosorbent assay (ELISA) technique. The statistical analysis was performed using SPSS 24. Overall, the matrix metalloproteinase-9 levels of the diabetic patients with chronic periodontitis were increased almost twofold (156.95 ± 29.80 ng/mL) compared to the levels in the controls (74.96 ± 6.32 ng/mL) (*p* < 0.001). Similarly, the periodontal parameters were far worse in diabetics with chronic periodontitis compared to the controls. The average gingivitis score for the test subjects was 78.45 ± 8.93%), compared to 29.32 ± 12.96% in the controls (*p* < 0.001). The mean probing pocket depth for the test group was 5.39 ± 0.60 mm, and, for the control group, it was 4.35 ± 0.31 mm (*p* < 0.001). For the test subjects, the average clinical attachment level was 5.86 ± 0.58 mm, and it was 4.66 ± 0.32 mm for the controls (*p* < 0.001). It was ascertained that there is a two-fold increase in the levels of salivary matrix metalloproteinase-9 in the test group compared to the control group. In addition, the level of periodontal apparatus destruction was greater in the test group. This proved that type-II diabetes mellitus influences the levels of matrix metalloproteinase-9 in humans and elevates them, causing further periodontal destruction.

## 1. Introduction

Periodontitis is known to be a chronic inflammatory disease that is known to cause the destruction of the supporting structures of the teeth, namely the periodontal ligament and alveolar bone. It has a very high prevalence rate, impacting negatively on the quality of life as well. A meta-analysis study [1] confirmed diabetes to be a major risk factor for chronic periodontitis, increasing the susceptibility of chronic periodontitis in diabetics by almost three-fold. The degree of hyperglycemia and severity of periodontitis are known to have a clear relationship with one another. There is an incomplete understanding about the mechanisms linking both, but there is known to be an involvement of the aspects of immune functioning, neutrophil activity and cytokine biology. However, recently, some evidence has emerged that explains the existence of a two-way relationship between diabetes and periodontitis, with diabetes known to increase the risk for periodontitis, and periodontal inflammation having a negative impact on glycemic control [2].

Both categories of diabetes, namely type I and type II, are associated with elevation in the levels of systemic markers of inflammation [3]. This elevation in diabetic inflammation contributes to both microvascular and macrovascular complications, and it has been determined that hyperglycemia may be involved in the activation of pathways that increase inflammation, oxidative stress and apoptosis [4]. In turn, periodontal tissue inflammation is increased by diabetes.

At the local and systemic levels, both periodontitis and diabetes mellitus are thought to share a common pathogenesis that involves an enhanced inflammatory response [5]. Recent theories have emerged, stating the role of an enhanced release of proinflammatory cytokines (interleukin-1Beta, interleukin-6, tumor necrosis factor alpha and matrix metalloproteinases), an altered receptor activator of nuclear factor kappa-Β ligand/osteoprotegerin (RANKL/OPG) ratio, an advanced glycation end-product/receptor for advanced glycation end-product (AGE) interactions, an increase in the leukocyte–endothelial cell interaction and an increased production of reactive oxygen, which all play pivotal roles in the two-way relationship between diabetes mellitus and chronic periodontitis [6], The presence of diabetes mellitus results in these complex changes, causing a modification of the local inflammatory reaction in the periodontium of patients with diabetes, which leads to a proinflammatory state in the microcirculation and gingival tissue.

Matrix metalloproteinases (MMPs) are a family consisting of 25 members, which are further categorized into six groups, based upon the sequence similarity, substrate specificity and domain organization. They are named gelatinases, collagenases, matrilysins, stromelysins and membrane-type MMPs. MMPs are a large family consisting of calcium-dependent zinc containing endopeptidases, having well- characterized structural and catalytic properties that are responsible for tissue remodeling and the degradation of the extracellular matrix (ECM), which is inclusive of gelatin, collagens, elastins, proteoglycans and matrix glycoprotein [7]. MMP production is induced during various physiological and pathological events. In periodontal ligaments (PDLs), MMPs are known to degrade the collagen of ECM, as well as to cause cytokine action and the activation of osteoclasts. The identification of different types of MMPs and their inhibitors in gingival crevicular fluid, saliva or blood may serve as biomarkers of chronic periodontitis, as well as therapeutic targets [8].

MMP-9 belongs to the gelatinase family. It plays several important functions, such as the degradation of the ECM, activation of interleukin-1Beta (IL-1β) and the cleaving of several chemokines [9]. An increase in the concentrations of salivary and serum MMP-9 and decrease in levels after periodontal therapy was demonstrated in several other studies, indicating the role of MMP-9 in periodontal diseases [10,11].

Periodontal diseases and diabetes mellitus are linked closely, and are highly prevalent chronic diseases, sharing a similar pathobiology. The inflammatory pathway is the major player in this association, and its importance is coming to light in recent times. Inflammatory markers can be targeted and studied to assess this relationship, and MMPs are crucial markers whose importance is being highlighted in recent studies as potential biomarkers. The primary MMPs of chronic periodontitis are MMP-8 and MMP-9. Studies have been carried out on salivary MMP-8 and crevicular MMP-2, which were known to serve as biomarkers of periodontal disease; however, there is a dearth of literature on how type-II diabetes mellitus (type-II DM) influences the levels of MMP-9 in chronic periodontitis patients [11,12].

In this study, the relationship of chronic periodontitis and type-II DM based on the levels of MMP-9 in saliva was determined by identifying whether the levels of MMP-9 are increased due to type-II DM in chronic periodontitis patients, in comparison to chronic periodontitis patients without type-II DM.

## 2. Results

A total of 60 consenting subjects participated in this study. The subjects were divided into test and control groups, each consisting of 30 members. A description of the test and control characteristics is provided in Table 1. The mean ages for the test group and controls were 50.73 ± 9.85 years and 48.07 ± 11.80 years (*p* > 0.05), respectively. The test group contained a marginally greater number of females, whereas the control group had a slight male majority. There were 13 males (43.3%) and 17 females (56.7%) in the test group. The control group consisted of 16 males (53.3%) and 14 females (46.7%) (*p* > 0.05). Based on ethnicity, there was a high prevalence of Malay population in both the test and control groups, the test group having 28 Malay (93.3%) and control group consisting of 26 Malay (86.7%). There were two Chinese (6.7%) in the test group and four Chinese (13.3%) (*p* > 0.05) in the control group.

As stated in Table 2, the mean plaque score for the test group was recorded as 33.78 ± 22.63%, and for the control group, it was 29.73 ± 26.50% (*p* > 0.05). The mean gingivitis score for the test group was recorded as 78.45 ± 8.93%, and for the control group, it was recorded as 29.32 ± 12.96% (*p* < 0.001). The mean probing pocket depth (PD) for the test group was calculated as 5.39 ± 0.60 mm, and for the control group, it was 4.35 ± 0.31 mm (*p* < 0.001). The mean clinical attachment level for the test group was 5.86 ± 0.58 mm, and it was 4.66 ± 0.32 mm (*p* < 0.001) for the control group.

It was observed that the MMP-9 concentrations of the diabetic subjects with chronic periodontitis showed an almost two-fold increase compared to the non-diabetic subjects. As shown in Table 3, the test group demonstrated a mean concentration of 156.95 ± 29.80 ng/mL, compared to a mean concentration of 74.96 ± 6.32 ng/mL (*p* < 0.001) in the control group.

## 3. Discussion

The two-way relationship between chronic periodontitis and diabetes mellitus has been cemented by numerous prior studies [2,13], each one being a risk factor for the other. This relationship basically relies on the inflammation pathway, which is a factor in the pathogenesis of both chronic periodontitis as well as diabetes mellitus. The inflammation process results in the release of certain mediators, which are the effector to bring about the changes in the normal healthy state of the cells or tissues and to cause them to become diseased. If these mediators can be controlled or if their effects can be toned down, this can result in a decrease or possible prevention of the harmful effects of a disease, in this case, being chronic periodontitis and/or diabetes mellitus.

The total number of subjects for the second study was 60:30 subjects each in the test and control group. This was in conjunction with the sample size for a similar study, in which, there were 23 subjects selected for type-II DM and chronic periodontitis and 26 systemically healthy subjects but with chronic periodontitis [14].

The difference in the mean ages of the test and control groups was not significant. The mean age for diabetics with chronic periodontitis was slightly greater than that of non-diabetics with chronic periodontitis. The results of this study suggest that type-II DM and chronic periodontitis have a relatively late onset in life, and are prevalent among the middle-age groups, but this finding cannot be generalized. The similarity in mean ages for both groups ensured homogeneity in results, with a minimization of bias due to age, as chronic periodontitis can be aggravated with increasing age.

There were slightly more females in the test group compared to males. On the other hand, in the control group, there was a slight majority of male subjects compared to females. However, overall, there was no significant difference in the male to female ratio in both groups. As per these findings, there is no difference in the prevalence of either type-II DM or chronic periodontitis in accordance with gender, though past literature suggests females have a greater genetic predisposition of type-II DM [15], whereas males are more prone towards developing chronic periodontitis [16]. The subjects were predominantly Malays, accounting for almost 90% of the total sample size. Other subjects were all Chinese. This can be explained by the fact that the total population of Malaysia consists majorly of the Malay race, followed by the Chinese group and then the Indians, with the rest following [17]. Specifically, in Kelantan, 95% of the total population is Malay, followed by Chinese, which is the second-most predominant race, accounting for almost 3% of the total population. The remaining population generally consists of Indians, which make up almost 2% of the total Kelantanese population [18].

Apart from the plaque score, all of the periodontal parameters were significantly higher in subjects with diabetes and chronic periodontitis compared to subjects with chronic periodontitis alone. These results prove that the severity of chronic periodontitis is markedly increased due to type-II DM, even when the oral hygiene status of the patients is more or less similar, and fair. A recent study conducted in Saudi Arabia was in line with our results, showing significantly higher levels of all periodontal parameters in chronic periodontitis subjects with diabetes compared to the control group [19]. Another study conducted in Italy in 2005 supported our results, showing a marked increase in the periodontal parameters of diabetics compared to the controls [20].

These results go on to signify that diabetes mellitus plays an important role in furthering the periodontal destruction of a patient, even when the oral hygiene status is fair. The periodontal status of diabetic subjects is worse than non-diabetic subjects with chronic periodontitis. From these results, it can be concluded that hyperglycemia worsens the periodontal status of subjects compared to normoglycemic individuals. The primary explanation for this is that chronic hyperglycemia increases the production of proinflammatory cytokines, such as IL-6 and MMPs, by human gingival fibroblasts, compared to normal glucose [21]. Another explanation for this result is that the interaction between advanced glycated end-products and their receptors is significantly higher in inflamed periodontal tissues with induced hyperglycemia than in normoglycemic individuals. This explanation was proved in a study conducted on rats in which diabetes and periodontitis were induced in the rats [22]. Another study reported that hyperglycemia increases the expression of Toll-like receptors (TLRs) in periodontal tissues, which contribute to a greater inflammatory response in hyperglycemic subjects and, resultantly, to periodontal disease as well [23].

This suggests that as the severity of hyperglycemia rises, the periodontal inflammatory response is also expected to increase. Another study suggested that diabetes results in changes in the function of immune cells, including neutrophils, monocytes and macrophages [24]. This will eventually predispose to chronic inflammation, progressive tissue breakdown and a diminished tissue repair capacity. This, in turn, causes chronic periodontitis to aggravate.

For the analysis of MMP-9 levels in the salivary samples, though there are many other diagnostic methods available, such as Western blotting, dentoAnalyzer, etc., our method of choice was ELISA. In comparison to the other options, ELISA is the most accurate quantitative test, whereas others are good options as qualitative tests [25]. In addition, ELISA was the chosen diagnostic method because of its high specificity and sensitivity, as it can detect inactive forms of MMP-9 in addition to the active forms, and can detect MMP-9 levels in the scale of picograms/mL [26].

As was the case with periodontal parameters, there was a significant increase in MMP- 9 levels in the saliva samples of the test group in comparison to the saliva samples of the control group. For the test group, the mean MMP-9 level was 156.95 ± 29.80 ng/mL. The mean MMP-9 level for the control group was 74.96 ± 6.32 ng/mL. A similar study conducted on gingival crevicular fluid (GCF) samples instead of saliva concluded the MMP-9 levels to be 17.1 ± 1.7 ng/dL for subjects with chronic periodontitis [27]. The MMP-9 levels for subjects with both type-II DM and chronic periodontitis came out to be 32.8 ± 11.1 ng/dL in another study [14]. Though the overall levels of MMP-9 were markedly lower than the results of our study, which might owe to the use of a different diagnostic fluid for analysis (GCF instead of saliva) and other factors, the overall increase in MMP-9 levels can be seen to be elevated by almost twice as much in subjects with type-II DM compared to non-diabetic chronic periodontitis subjects, which is synonymous with our findings.

Our results also depict an almost two-fold increase in the MMP-9 levels of type-II DM subjects with chronic periodontitis compared to the control group. This signifies that type-II DM causes increased levels of MMP-9 to be released in its inflammation that are almost twice as much as the levels released in the inflammation caused by chronic periodontitis only. As a result, it can be estimated that these elevated levels of MMP-9 in type-II DM are one of the important factors responsible for the periodontal destruction in diabetic subjects and, if controlled, the severity of chronic periodontitis may be reduced in diabetic subjects.

There are some limitations of this study (due to time limitations and limited research grant funding) that may be overcome for future investigations. A larger sample size would provide greater depth toward the current hypothesis and results. The subjects only consisted of a majority of one type of race, which was Malay, and there was a very low frequency of Chinese population. Representation from more Chinese and other races would provide a diverse representation in the results. Apart from the ELISA assay, there are other methods for the measurement of MMP levels that could have been constituted; for example, Western blot, dento-analyzer, time-resolved fluorescent immunoassay, etc. The relationship of hyperglycemia with MMP-9 levels can be assessed if random glucose levels are recorded for each subject together with their saliva samples collection. This could provide a deeper understanding on this topic.

## 4. Materials and Methods

### 4.1. Study Design

This was an observational, analytical, case-control study carried out at School of Dental Sciences, Universiti Sains Malaysia (USM) Health Campus, Kelantan.

### 4.2. Ethical Consideration

The ethical approval was obtained from The Human Research Ethics Committee of USM (JEPeM) with ID number USM/JEPeM/17030192.

### 4.3. Sample Size Estimation

The sample size was calculated by using reference values from a study carried out on levels of MMP-9 in gingival crevicular fluid (GCF). The level of MMP-9 in GCF of chronic periodontitis patients turned out to be 17.1 ± 1.7 ng/dL [23], and that of patients with both chronic periodontitis and type-II DM was 32.8 ± 11.1 ng/dL [14]. This meant 1.9 times increase in MMP-9 level due to type-II DM. Assuming the same results to stay valid for saliva and taking the level of MMP-9 in saliva of chronic periodontitis patients to be 272.2 ± 256.1 ng/mL [28], we assumed that MMP-9 levels should reach around twice as much in patients with both chronic periodontitis and type-II DM. With these numbers, the following calculations were carried out, and sample size calculated accordingly:

Type I error probability (α) = 0.05;
Power = 0.8 (80%);
Difference in population means (δ) = 200 (rounded off to nearest hundred);
Standard deviation (σ) = 256.1 (largest value of the two populations selected);
Ratio of control to experimental patients (m) = 1.

PS Power and Sample Size Calculations version 3.0 was used with the above values to calculate the sample size, which came out to be 27. Taking account of the 10% dropout rate of patients, the sample size was rounded off to 30 patients in each group.

### 4.4. Data Collection

The data for this study were collected in a period of 6 months, starting from October 2017 until March 2018. Laboratory analysis was completed in a month’s time, from April to May 2018.

The sampling method used for this study was the probability, clustered sampling method. A total of 60 patients were recruited, divided equally among the control and the test group. The control group consisted of patients diagnosed with chronic periodontitis only (and no systemic diseases), whereas the test group constituted patients diagnosed with both type-II DM and chronic periodontitis. The participants were distributed into test and control groups after assessing the medical history and measuring the random blood glucose levels (for control group) or checking their HbA1c levels (for test group). Those with HbA1c ≥ 6.5% were selected only, as they are proven diabetics.

All information, such as patients’ socio-demographic data (age, gender and race), medical history and periodontal parameters (plaque score, gingival bleeding index, probing pocket depth and clinical attachment level), were recorded.

All of the selected patients were adults aged between 30–70 years. Only the patients diagnosed with mild to moderate chronic periodontitis, i.e., with a mean probing pocket depth >3 and <5 mm (mild) and ≥5 and <7 mm (moderate) were selected. For the test group, only the patients clinically diagnosed with type-II DM at least 6 months prior and with the above-mentioned criteria of chronic periodontitis were selected [29].

Pregnant and lactating women, patients with acute or chronic medical disorders and those who had undergone periodontal therapy at least 3 months prior to data collection were excluded.

The patients were taken through a complete periodontal checkup. The inclusion and exclusion criteria were applied to screen out eligible participants. Participants were informed in detail about the research and all their questions and concerns were addressed beforehand. A written and signed consent was obtained from all of the participants.

The subjects were subjected to the following procedures, in order:

1. Recording of periodontal parameters;
2. Collection of saliva;
3. Collected saliva transported to the laboratory and stored in −80 °C freezers until further laboratory analysis.

The collected saliva samples were later analyzed in the laboratory using enzyme linked immunosorbent assay (ELISA) test, using Human Matrix Metalloproteinase-9 ELISA Kit (Elabscience, Houston, TX, USA).

#### 4.4.1. Periodontal Parameters

The periodontal parameters recorded were:

1. Plaque Score (O’Leary, 1972)
  Four surfaces of each tooth (facial/buccal, mesial, distal, palatal/lingual), excluding all third molars, were checked for presence of plaque, marking any surface that had plaque presence on the chart. This was divided by the total number of surfaces checked (total number of teeth present × 4) and multiplied by one hundred. The resulting calculation was the plaque score of the patient, given as a percentage [30].
2. Gingival Bleeding Index (Ainamo and Bay, 1975)
  Similar to plaque score calculation, four surfaces of each tooth (facial/buccal, mesial, distal, palatal/lingual), excluding all third molars, were checked for bleeding on probing, marking any surface that showed bleeding on the chart. This was divided by the total number of surfaces checked (total number of teeth present × 4) and multiplied by one hundred. The resulting calculation was the gingivitis score of the patient, given as a percentage [31].
3. Probing Pocket Depth (PPD)
  The periodontal pocket depth is defined as the distance between the gingival margin and the bottom of the probable pocket to the nearest whole millimeter [32]. Using the Michigan O periodontal probe, there were 6 measurements taken for each tooth: 3 on the facial/buccal side (mid-, mesio- and disto-) and 3 on the lingual/palatal side (mid-, mesio- and disto-) [33].
4. Clinical Attachment Level (CAL)
  Whereas a periodontal pocket is the distance from the base of the pocket to the gingival margin, clinical attachment level is the distance from the base of the pocket to a fixed point on tooth, usually kept as the cemento-enamel junction (CEJ). Two measurements are used to calculate the CAL: the probing depth and the distance from the gingival margin to the CEJ. Taken together, the probing depth plus the distance from the gingival margin to the CEJ comprises the clinical attachment level [34].

#### 4.4.2. Inter-Examiner Reproducibility

The inter-examiner reliability on and reproducibility of the measurement of probing pocket depth (PPD) and clinical attachment level (CAL) were ascertained before the start of data collection. Repeated measurements were carried out on 120 sites of teeth for different patients between two examiners. The intraclass correlation coefficient (ICC) test was used to analyze the measured values. The correlation coefficients derived were 0.96 and 0.88 for PPD and CAL, respectively.

ICC of 0 indicates no reliability, whereas ICC of 1.0 indicates perfect reliability, and the effect of measurement error becomes minimal as ICC goes above 0.8 [35].

#### 4.4.3. Saliva Samples Collection

After recording the clinical parameters described above, the saliva samples necessary for estimation of MMP-9 were taken from subjects of both the groups. Stimulated saliva was collected as previous studies have demonstrated that stimulated saliva flow rate is greater than unstimulated method of collection [36]. Subjects were asked to smell a citrus fruit (lemon or orange) prior to sample collection. This stimulates the salivary glands to produce saliva in greater amounts than normal. Then, the subjects were given distilled drinking water and asked to rinse their mouth out well for 1 min. The subjects were then asked to expectorate the water. Five minutes after this oral rinse, the subjects were asked to spit into a 15 mL sterile falcon tube. Subjects were instructed to allow saliva to pool at the bottom of the mouth and passively flow into the saliva collecting tube. These samples were stored at −80 °C till further analysis.

#### 4.4.4. Preparation of Saliva Samples

Before running the ELISA test, the saliva samples were prepared for the assay. All of the saliva samples were taken out from the −80 °C deep freezers and thawed for 1–2 h. After the samples thawed completely, they were put in the centrifuge at 1000× *g* for 20 min at 5 °C to remove cell debris. Then, the supernatant was removed and stored in small aliquots of 1.5 mL each in Eppendorf tubes (Eppendorf AG, Hamburg, Germany). After few pre-runs, it was determined that the MMP-9 levels in saliva were greater than the detection range of Human MMP-9 ELISA Kit (Elabscience, Houston, TX, USA). Therefore, according to the pre-run results, a dilution factor of 1:100 was established for the saliva samples in order to attain the MMP-9 concentration within the detection range of the kit. To make these dilutions, 198 µL of reference standard and sample diluent was added to every 2 µL part of saliva sample, and 200 µL of diluted saliva aliquot was prepared for each sample. These aliquots were then stored at −20 °C overnight until laboratory analysis. The remnant samples of saliva were discarded and destroyed appropriately.

### 4.5. Preparation of Reagents

Firstly, the wash buffer solution was prepared. A total of 30 mL of concentrated wash buffer was diluted with distilled water to prepare 750 mL of wash buffer.

Then, the standard working solution was prepared. The standard was centrifuged at 10,000× *g* for 1 min, and 1.0 mL of reference standard and sample diluent was added to it. It was left standing for 10 min, turning it upside down several times during this period. After it dissolved fully, it was mixed thoroughly again with a pipette. This reconstitution produced a working solution of 2000 pg/mL. Serial dilutions are made from this working solution.

For serial dilutions, seven Eppendorf tubes, each of 1.5 mL capacity, were taken and filled with 500 µL of reference standard and sample diluent each. Then, 500 µL of the 2000 pg/mL working solution was pipetted into the first tube and mixed to produce a 1000 pg/mL working solution. Then, a further 500 µL was pipetted from this 1000 pg/mL working solution and mixed into the next tube. The same procedure was repeated until the last tube. As the last tube is regarded as a blank, solution from former tube was not pipetted into it. This method of serial dilutions gave the following working solution concentrations: 2000 pg/mL, 1000 pg/mL, 500 pg/mL, 250 pg/mL, 125 pg/mL, 62.5 pg/mL, 31.25 pg/mL and 0 pg/mL (blank).

Then, the biotinylated detection antibody and concentrated HRP conjugate working solution was prepared. The required amount for each is 100 µL/well. Therefore, it needs to be diluted from 100× to 1× working solution and should be enough for each well. As a precaution, 100–200 µL extra solution was prepared. The dilution equation M1V1 = M2V2 was used for this calculation, where

M1 = concentration in molarity of concentrated substance (100), V1 = volume of concentrated substance (unknown), M2 = concentration in molarity of diluted substance (1) and V2 = volume of diluted substance (9800).

As there are 96 wells in the plate and 100 µL was required for each well, 9600 µL of total volume was required for the working solution. Adding the extra 200 µL, it came out to be 9800 µL. Using the dilution equation, V1 came out to be 98 µL. Therefore, to prepare the working solutions for biotinylated detection antibody and concentrated HRP conjugate, 98 µL of concentrated solution was required and 9702 µL (9800–98) of diluent was required to produce the 1× working solution for each. The stock tubes for both were centrifuged at 10,000× *g* for 1 min before the dilution was made.

### 4.6. Protocol for ELISA Assay

Standard working solution of different concentrations was added to the first two columns, each concentration of solution being added into two wells side by side and 100 µL for each well. Similarly, saliva samples were added to the remaining wells, each sample added into two wells side by side (duplication) and 100 µL for each well. The plate was covered with plate sealer and put for incubation at 37 °C for 90 min.

After this, liquid from each well was removed, and 100 µL of biotinylated detection antibody working solution was immediately added to each well. Plate was again covered with sealer, gently mixed up and put into incubation at 37 °C for 1 h.

Then, solution from each well was decanted and 300 µL of wash buffer was added to each well. The wells were soaked for 1–2 min and then decanted, patting the plate dry against a clean absorbent paper. This washing step was repeated thrice in total. After the washing, 100 µL of HRP conjugate working solution was added to each well. The plate was covered again with sealer and incubated at 37 °C for 30 min.

When this time had elapsed, the solution from each well was again decanted and the wash process with wash buffer was repeated five times. Then, 90 µL of substrate reagent was added to each well. This step was conducted in a dark environment so as to protect the plate from light, the substrate reagent being light-sensitive. Plate was covered with a new sealer and wrapped in aluminum foil. Then, it was again incubated at 37 °C for 15 min. The plate was observed in incubation after every 5 min for color changes. After 15 min, when ample color change had occurred in all of the wells, 50 µL of stop solution was added to each well, ensuring that stop solution was added to each well in the same order as that of substrate reagent addition.

After this, the plate was inserted into the Varioskan Flash Multimode Reader (Thermo Fisher Scientific, Waltham, MA, USA) and the optical density (OD) value of each well was determined, keeping the wavelength setting at 450 nm. The blank solution well was the background for each kit, and its OD value was subtracted from the OD value derived for each well to obtain the corrected OD readings.

**Figure 1:**
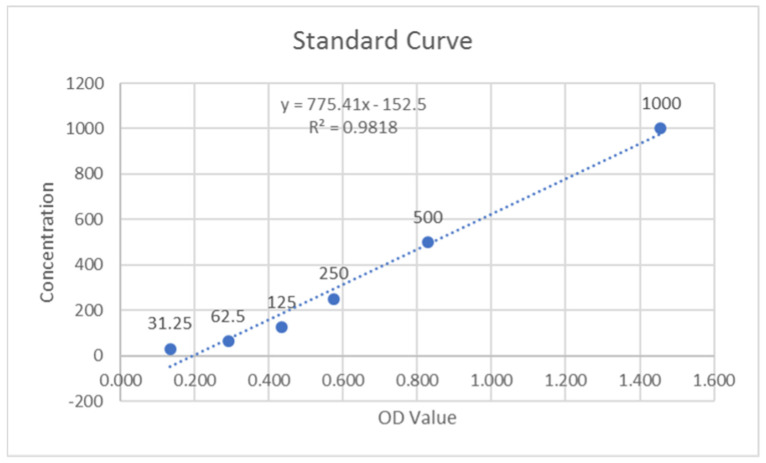
Standard curve of MMP-9 for control group.

**Figure 2:**
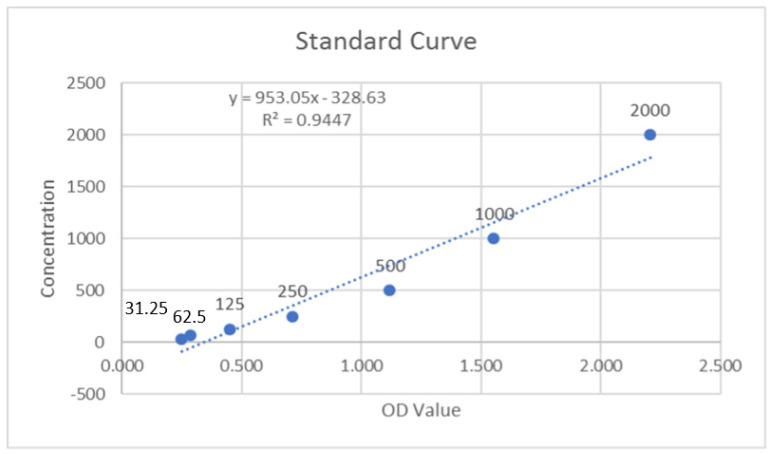
Standard curve of MMP-9 for test group.

The optical density (OD) values of the duplicate samples were analyzed, and the mean value was calculated. These values were inclusive of the background subtraction. The MMP-9 standards were analyzed, and their linear graphs were plotted for control and test groups (Figure 1 and Figure 2). These graphs were used to assess the MMP-9 concentrations of samples by using their respective equations. As there was a dilution factor of 1:100 used for the saliva samples, the resulting concentrations were multiplied by 100 to achieve the corrected concentrations. The units of concentration, which were initially in pg/mL, were converted to ng/mL after this multiplication.

**Figure 3:**
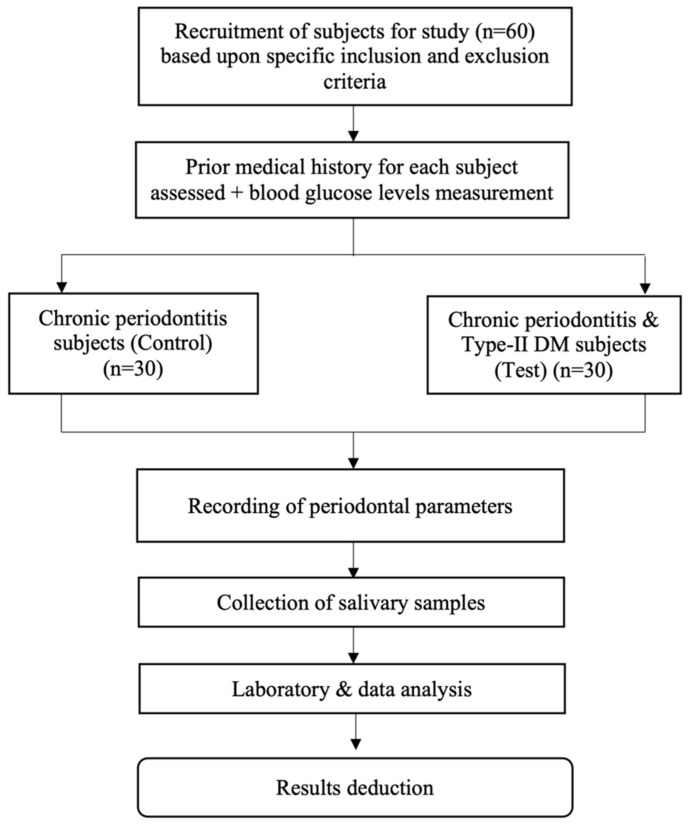
Flowchart of the study.

A complete flowchart of the study protocol is depicted in Figure 3.

### 4.7. Data and Statistical Analysis

All data were analyzed using SPSS version 24.0 statistical software (IBM Corp., NY, USA). Continuous data were summarized as mean with standard deviation (SD), whereas discrete data (categorical) as mean percentage with SD. Continuous variables were compared by analysis of variance (ANOVA) and the significance of mean difference between the groups was carried out by independent *t*-test. Categorical variables were also compared by independent *t*-test. *p* value of 0.05 was taken as indicative of statistical significance.

## 5. Conclusions

There was an almost two-fold increase in the average salivary MMP-9 levels of chronic periodontitis patients with type-II DM compared to the chronic periodontitis patients alone. This shows that type-II DM influences the expression of salivary MMP-9, which, in turn, holds the ability to aggravate the periodontal apparatus degradation in type-II DM patients.

Another deduction from this study was that the values for all of the periodontal parameters were significantly higher in chronic periodontitis patients with type-II DM compared to the chronic periodontitis patients alone, depicting that the periodontal status of chronic periodontitis patients suffering from type-II DM was worse than non-diabetic chronic periodontitis patients. This signifies that hyperglycemia is a root cause of an exaggerated inflammatory response that causes an increase in the release of proinflammatory cytokines, causing further degradation of the periodontal apparatus.

## Figures and Tables

**Table 1:** Demographic analysis of subjects.

Demographic Characteristics	Subjects		*p* Value *
	Type II Diabetes Mellitus and Chronic Periodontitis (Test) (*n* = 30)	Chronic Periodontitis (Control) (*n* = 30)	
Age [mean ± SD, in years]	50.73 ± 9.85	48.07 ± 11.80	0.11
Gender			0.439
Male, *n* (%)	13 (43.33%)	16 (53.33%)	
Female, *n* (%)	17 (56.67%)	14 (46.67%)	
Race			0.671
Malay, *n* (%)	28 (93.33%)	26 (86.67%)	
Chinese, *n* (%)	02 (6.67%)	04 (13.33%)	

* Independent *t*-test used; values represent the means and standard deviations or numbers (%) of subjects/sites.

**Table 2:** Periodontal parameters of subjects.

Clinical Parameters (mean ± SD)	Subjects		*p* Value *
	Type II Diabetes Mellitus and Chronic Periodontitis (Test) (*n* = 30)	Chronic Periodontitis (Control) (*n* = 30)	
Plaque score [%]	33.78 ± 22.63	29.73 ± 26.50	0.071
Gingivitis score [%]	78.45 ± 8.93	29.32 ± 12.96	<0.001
Probing pocket depth [mm]	5.39 ± 0.60	4.35 ± 0.31	<0.001
Clinical attachment level [mm]	5.86 ± 0.58	4.66 ± 0.32	<0.001

* Independent *t*-test used; values represent the means and standard deviations or numbers (%) of subjects/sites.

**Table 3:** Comparison of the levels of MMP-9 in salivary samples.

Biomarkers (Concentration, mean ± SD)	Subjects		*p* Value *
	Type II Diabetes Mellitus and Chronic Periodontitis (Test) (*n* = 30)	Chronic Periodontitis (Control) (*n* = 30)	
Salivary MMP-9 [ng/mL]	156.95 ± 29.80	74.96 ± 6.32	<0.001

* Independent *t*-test used; values represent the means and standard deviations of subjects.

## Data Availability

Apart from the personal data of the involved subjects, which were kept confidential, all the data collected and analyzed are mentioned in the manuscript.

## Sample Availability

Samples of the compounds were discarded appropriately after analysis and are not available from the authors.
